# Setting proficiency standards for simulation-based mastery learning of short antegrade femoral nail osteosynthesis: a multicenter study

**DOI:** 10.2340/17453674.2024.40812

**Published:** 2024-05-30

**Authors:** Amandus GUSTAFSSON, Jan D RÖLFING, Henrik PALM, Bjarke VIBERG, Søren GRIMSTRUP, Lars KONGE

**Affiliations:** 1 Orthopaedic Department, Slagelse Hospital, Region Zealand, Slagelse; 2 Copenhagen Academy for Medical Education and Simulation, Rigshospitalet, Copenhagen; 3 Department of Clinical Medicine, Faculty of Health Science, University of Copenhagen, Copenhagen; 4 Department of Orthopaedics, Aarhus University Hospital, Aarhus; 5 MidtSim, Corporate HR, Central Denmark Region, Aarhus; 6 Orthopaedic Department, Bispebjerg Hospital, Region H, Copenhagen; 7 Orthopaedic Department, Odense Hospital, Region Syd, Odense, Denmark

## Abstract

**Background and purpose:**

Orthopedic trainees frequently perform short antegrade femoral nail osteosynthesis of trochanteric fractures, but virtual reality simulation-based training (SBT) with haptic feedback has been unavailable. We explored a novel simulator, with the aim of gathering validity evidence for an embedded test and setting a credible pass/fail standard allowing trainees to practice to proficiency.

**Patients and methods:**

The research, conducted from May to September 2020 across 3 Danish simulation centers, utilized the Swemac TraumaVision simulator for short antegrade femoral nail osteosynthesis. The validation process adhered to Messick’s framework, covering all 5 sources of validity evidence. Participants included novice groups, categorized by training to plateau (n = 14) or to mastery (n = 10), and experts (n = 9), focusing on their performance metrics and training duration.

**Results:**

The novices in the plateau group and experts had hands-on training for 77 (95% confidence interval [CI] 59–95) and 52 (CI 36–69) minutes while the plateau test score, defined as the average of the last 4 scores, was 75% (CI 65–86) and 96% (CI 94–98) respectively. The pass/fail standard was established at the average expert plateau test score of 96%. All novices in the mastery group could meet this standard and interestingly without increased hands-on training time (65 [CI 46–84] minutes).

**Conclusion:**

Our study provides supporting validity evidence from all sources of Messick’s framework for a simulation-based test in short antegrade nail osteosynthesis of intertrochanteric hip fracture and establishes a defensible pass/fail standard for mastery learning of SBT. Novices who practiced using mastery learning were able to reach the pre-defined pass/fail standard and outperformed novices without a set goal for external motivation.

Osteosynthesis of a proximal femoral fracture (PFF) is a common orthopedic procedure that must be conducted acutely. Hence, surgical procedures for PFF must often be mastered by trainees early in their careers, but evidence suggests that surgery by surgeons in training can be associated with higher reoperation rates [1,2].

A proposed method to mitigate increased risk is simulation-based training (SBT), as it is associated with higher learning outcomes than other instructional modalities [3]. In a national needs assessment of SBT, osteosynthesis for PFF was ranked 2nd of 33 prioritized procedures within orthopedic and trauma surgery [4].

Simulators exist for cannulated screws, Hansson pins, and sliding hip screw (SHS) [5-7]. However, a short antegrade nail is a frequent choice for osteosynthesis of extracapsular PFF in many countries [8], but no virtual reality simulator with force feedback for this procedure has been described.

Consequently, in cooperation with Swemac Education, we developed the software for training short antegrade femoral nail osteosynthesis on their simulator platform TraumaVision (Swemac Simulation AB, Linköping, Sweden).

Mastery learning allows all trainees to continue training until they reach a pre-defined pass/fail standard. Evidence suggest that this approach has a higher efficacy than other learning approaches [9]. Credible pass/fail standards are necessary to implement mastery learning training programs and a rigorous validation process using a contemporary framework is mandated [10]. Though validity evidence may make a test credible, it gives little or no clue as to what level of training is necessary to reach proficiency for the trainee before performing the procedure on patients under supervision [11]. Also, pass/fail standards are of little value if they are unobtainable for a sizable proportion of the trainees. Hence, exploring the consequence on the intended target is a paramount step when establishing credible pass/fail standards.

The objectives of this study were to examine validity of simulator test scores in relation to surgical experience with the procedure, to establish a credible pass/fail standard for trainee proficiency and explore the consequences of this standard.

## Methods

The “Reporting guidelines for health care simulation research: extensions to the CONSORT and STROBE statements” was followed.

The study was conducted from May 2020 to September 2020 in a collaboration between 3 Danish simulation centers: Corporate HR, MidtSim – Central Denmark Region, SimC – Odense University Hospital, and Copenhagen Academy for Medical Education and Simulation – Copenhagen University Hospital Rigshospitalet (CAMES). We established validity evidence for the test according to the validity framework suggested by Messick, which includes 5 different sources of validity evidence: content, response process, internal structure, relations to other variables, and consequences [12].

**Figure 1:**
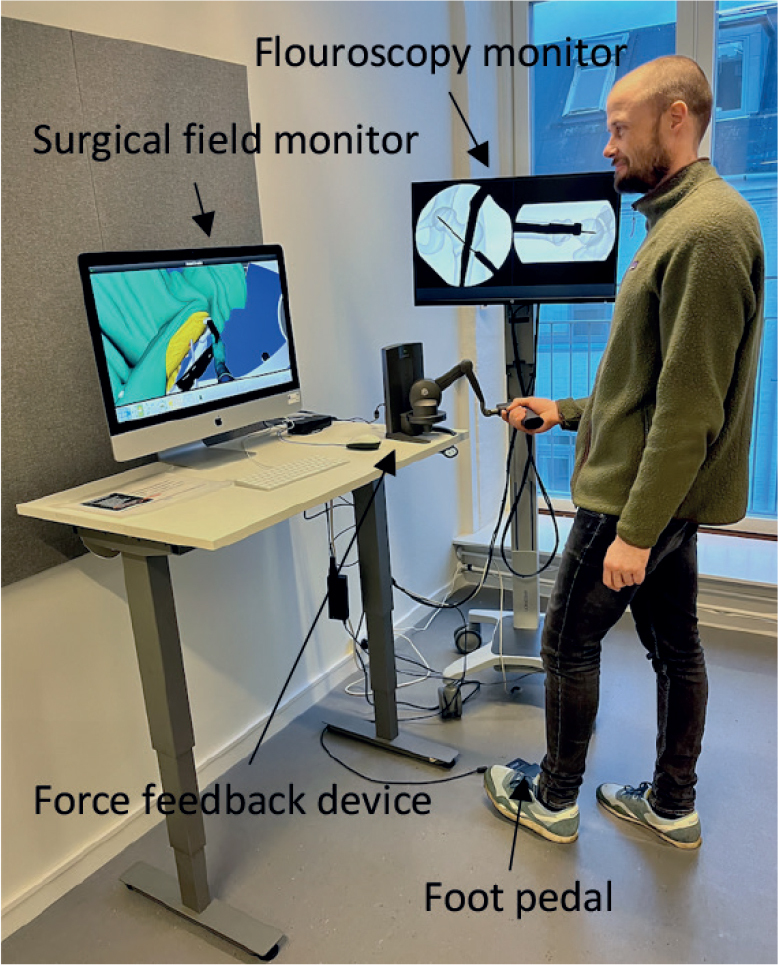
The setup of the TraumaVision simulator.

We used novel software for short antegrade femoral nail osteosynthesis on Swemac TraumaVision. The simulator consists of a computer with 2 monitors, a force feedback device (Phantom Omni or Touch X, 3D Systems, Rock Hill, SC, USA) that mimics the surgical tools and generates haptic feedback, and a computer. The movements are visualized on 1 of the monitors. The simulated fluoroscopy is administered by a foot pedal and can be displayed in either a standard anterior–posterior or lateral image on the other monitor (Figure 1).

### Development of the test

The simulator tracks several metrics during the simulation training including time spent, number of fluoroscopy images taken, entry point in the greater trochanter, lag screw placement etc. Prior to testing, 2 specialists in orthopedic trauma surgery (AG and JDR) selected metrics and acceptable values to what was deemed clinically relevant (Table 1). The simulator computes a combined score, which is a percentage of the maximum score based on the selected metrics. The metrics can be negative if the performance is far from the acceptable interval. Consequently, it is possible to achieve a negative combined score if the overall performance is exceptionally poor.

**Table 1:** Parameters and acceptable intervals for scoring in TraumaVision antegrade femoral nail simulator

Parameter	Unit	Acceptable interval
Total time	s	0–300
Total fluoroscopy time	s	0–300
Total no. of fluoroscopy images	n	0–40
Nail angle	°	120–130
Entry point (anterior/posterior)	mm	–2.0 to 3.0
Entry point (lateral/medial)	mm	–3.0 to 2.0
K-wire for opening reamer depth	mm	80–300
K-wire distance to joint surface	mm	1.0–3.0
Step drill distance to joint surface	mm	5.0–10.0
Lag screw distance to joint surface	mm	5.0–10.0
Lag screw length outside cortex	mm	0.0–2.8
Screwdriver handle—targeting device angle	°	0.0–10.0
Lag screw tip distance below center	mm	0.0–15.0
Lag screw tip distance to center	mm	0.0–3.0
Final set screw rotation	°	–110 to –70
Tip–apex distance	mm	10.0–20.0
Probability of cut out	%	0.0–1.8
Locking screw drill tip length outside cortex	mm	0.0–10.0
Locking screw tip length outside cortex	mm	2.0–4.0
Locking screw head distance to lateral cortex	mm	0.0–1.5

### Gathering test data from participants training to plateau

Previously published data on novice/expert comparisons of other simulations of PFF osteosynthesis utilizing the same simulator [6] found a plateau score mean of 85% (standard deviation [SD] 7.7) for the novices and 92% (SD 4.0) for the experts. To reduce the number of experts needed, we employed a 1.5:1.0 ratio when calculating the sample size, which resulted in 14 novices and 9 experts with an alpha of 0.05 and power of 0.80 [13].

**Figure 2:**
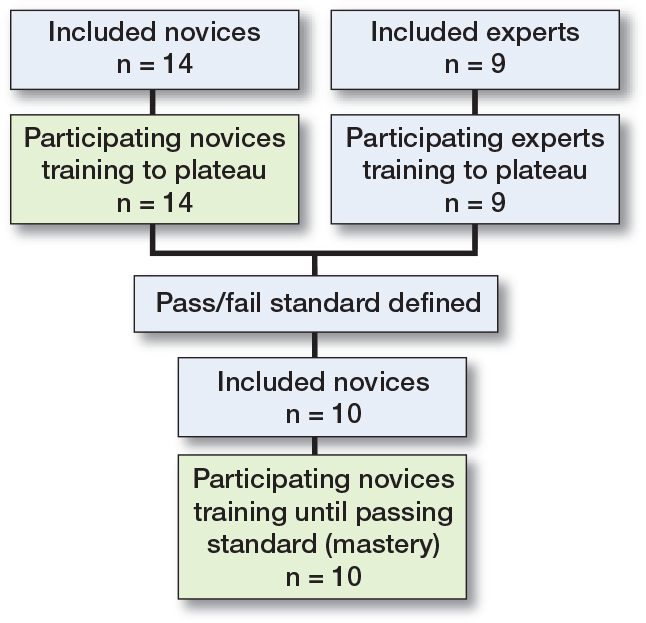
Flowchart of study participants.

Novices (n = 14) in their 1st year of specialization or interns employed in an orthopedic department and experts (n = 9) who were specialists in orthopedic trauma surgery participated in the study. Previous training on the simulator during the last 6 months was an exclusion criterion for both groups and more than 10 performed osteosyntheses of PFF of any type was an exclusion criterion for the novices (Figure 2).

All participants were introduced to the simulator and the correct operation technique prior to training. An orthopedic surgeon (authors JDR, BV, and AG) experienced in hip fracture surgery conducted a standardized introduction including a demonstration of the simulated procedure at each center. After completing the introduction, the participants completed a warm-up session. Subsequently, the participants trained individually and received simulator-generated feedback after each nailing procedure. A trained simulator assistant was available and helped the participants to interpret the simulator-generated feedback. The simulator assistant was not blinded as to whether the participant was a novice or expert. Each participant had to complete at least 6 attempts and continue to train until reaching the plateau phase indicated by 3 consecutive attempts without improvement.

### Gathering test data from mastery learning participants

Data collected from the expert group was used to create a pass/fail standard defined as the average of the experts’ 4 last attempts (i.e., their plateau score). A new cohort of novices (n = 10) in their 1st year of specialization were included in the 2nd part of the study. The size of the cohort was pragmatically determined based on feasibility of recruitment. Previous training on the simulator during the last 6 months or more than 10 performed osteosyntheses of PFF of any type were exclusion criteria (Figure 2). The training setup was an exact replica of that previously described with the exception that the novices were told they had to achieve the pass/fail standard to pass the test. The participants trained until they achieved the pass/fail standard.

### Statistics

Test/retest reliability of the last 2 trials on the simulator was assessed using the 2-way mixed consistency intra-class correlation coefficient (ICC). Levine’s tests were performed and independent samples t-tests with equal variances assumed/not assumed as appropriate were used to (i) compare the performance of the novice and expert groups for variables with normal distribution and (ii) compare continuous data for variables for the novice groups. Fisher’s exact test was used to compare categorical data for variables for the novice groups. For comparison of performance of the novice and expert groups for variables with non-normal distribution, a bootstrapped independent samples t-test was used. The plateau score is defined as the average of the participants’ last 4 scores. The mean plateau score distribution of the novices training to plateau and expert groups was plotted using the contrasting groups method [14]. The intersection between the 2 groups was compared with the pass/fail mastery standard, which was set at the mean plateau score of the experts. The statistical analysis was performed using SPSS version 25 (IBM Corp, Armonk, NY, USA). Differences in metrics were considered statistically significant when the P-value is < 0.05. For the results, 95% confidence intervals (CI) were used.

### Ethics, funding, and potential conflicts of interests

Ethical approval was obtained prior to commencement of the study from the Regional Ethical Committees of the Capital Region and Central Denmark Region in the form of exempt letters no. H-19068316 and 251/2016, respectively. The participants gave informed consent and could opt to drop out at any time. There was no external funding for the study. None of the authors have any competing interests to declare. Complete disclosure of interest forms according to ICMJE are available on the article page, doi: 10.2340/17453674.2024.40812

## Results

**Figure 3:**
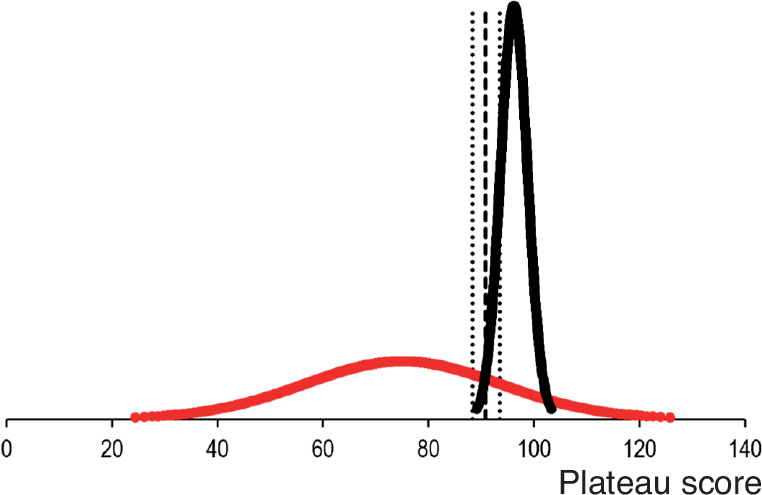
Distribution of plateau scores for novices (red) and experts (black). Using the contrasting groups method, a pass/fail standard of 91% (95% confidence intervals 88–94, dotted lines) can be determined from the intersection of the distributions (dashed line).

**Figure 4:**
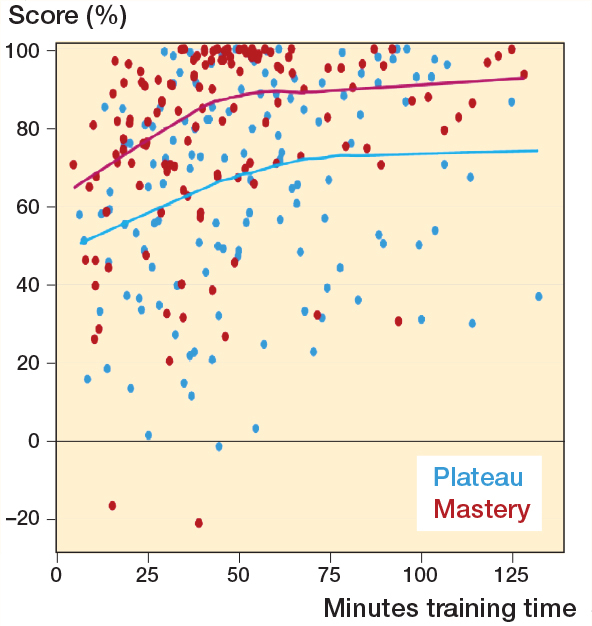
Scatterplot of test scores versus time spent training with average lines for novices training to plateau (blue) and novices training to mastery (red).

All included participants met the inclusion criteria, and no participants were excluded. The distribution of participants per institution is shown in Table 2. The 2 novice groups were similar as to age, sex, hand dominance, time working in an orthopedic department, number of antegrade short femoral nailings performed prior to participation, and previous experience with SBT (Table 3). The experts and novices in the plateau group had hands-on training for 52 (CI 36–69) and 77 (CI 59–95) minutes while the plateau score was 96% (CI 94–98) and 75% (CI 65–86) respectively. The contrasting groups method established a pass/fail cutoff at 91% (CI 88–94) (Figure 3). However, all novices in the mastery learning group (n = 10) were able to reach a pass/fail mastery standard of 96%. The hands-on training time for the novices in the mastery learning group was 65 minutes (CI 46–84). The novices training to plateau made on average 9 (CI 7–11) attempts while the novices training to mastery used 13 (CI 10–17) attempts to reach the pass/fail standard. Figure 4 illustrates the relationship between scores and time spent training for the 2 novice groups. The test–retest reliability between the 2 last attempts was 0.80 (CI 0.60–0.91). Sources of evidence in Messick’s validity framework are summarized in Table 4.

**Table 2:** Distribution of participants

Institution	Expert	Novices plateau	Novices mastery
MidtSim, Aarhus University Hospital	6	7	6
SimC, Odense University Hospital	–	2	1
CAMES, Rigshospitalet, Copenhagen	3	5	3
Total	9	14	10

**Table 3:** Demographics and previous experience for novice and expert groups

Group	n	Mean age (SD)	Male/female	Dominant hand (R/L)	Mean OE/YSS (SD)	Mean SP (SD)	Previous simulation
Novices plateau	14	30.1 (5.0)	8/6	12/2	6.9 (7.0) months	0.9 (2.2)	11
Novices mastery	10	29.6 (1.3)	8/2	8/2	6.1 (4.0) months	1.4 (1.6)	8
Experts	9	40.3 (5.3)	7/2	7/2	4.0 (3.3) years	–	1

R/L = right/left. OE = months of orthopedic employment (novices), YSS = years since specialization (experts). SP = supervised procedures.

**Table 4:** Sources of evidence in this study in relation to Messick’s validity framework

Source of evidence	Question related to each source of evidence	Validity evidence
Content	*Does the content of the test reflect the construct it is intended to measure?*	2 specialists in orthopedic trauma surgery selected metrics and acceptable values deemed clinically relevant (Table 1).
Response process	*What has been done to reduce bias?*	The participants received a standardized instruction and were tested in the same setting. The simulator provides objective metric scores.
Internal structure	*Is the test score reliable?*	Strong test–retest reliability measured by ICC.
Relationship with other variables	*Is there a correlation between performance score and a recognized measure of competency?*	Experts statistically significantly outperformed novices on plateau test score.
Consequences	*What are the consequences of the pass/fail standard?*	All novices in the mastery learning group were able to achieve the pass/fail standard based on experts’ average performance.

## Discussion

We developed a standardized test of short antegrade nail osteosynthesis of trochanteric hip fracture based on a virtual-reality simulator. Furthermore, we gathered evidence of validity for the test according to Messick’s 5 sources. The study demonstrates that the test for a novel simulator for training osteosynthesis of PPFs can discriminate between the target training group (novices) and traumatology specialists (experts). When compared with novices training to plateau, experts attain a much higher score while spending less time, though not statistically significant, in doing so. This infers supporting validity evidence of the test for assessment of competence for novices in the simulated setting.

The contrasting groups’ standard setting method (CGM) is a widely recognized and adapted tool to establish a pass/fail standard [14] for procedural performance in health profession education and this study indicates a pass/fail score of 91% (Figure 3). The method is a statistical algorithm that conveys an estimated score which allows as few novices and as many experts as possible to pass the test. However, we have previously expressed concern that the pass/fail score is too low to guide the decision on when novices are ready to move from the simulated setting to perform procedures on patients under supervision [6]. First, several studies indicate that novices terminate their training before achieving optimal performance when performing self-directed training [11,15]. Consequently, there is an inherent risk that the novices’ performance data is relatively low, which will result in a lower pass/fail score using the CGM. Second, the purpose of establishing a credible pass/fail standard for competence in the simulated setting for surgeons in training is not to find the score that makes most experts pass the test, but that the trainees are trained to the highest level of competence they can achieve, albeit within a reasonable timeframe. Hence, we decided to raise the pass/fail standard to the level of the experts’ average score of 96% to gain a real mastery competence standard and continued to explore the consequences of this pass/fail standard in terms of practical feasibility.

Interestingly, we found that all novices in the mastery learning group were able to obtain the experts’ average score with no other intervention than that they were instructed to obtain the specific score. Studies have indicated that novices in the simulated setting in some cases can obtain expert-level scores [16-17]. More surprisingly, we found that the novices in the mastery learning group obtained expert-level scores without using more hands-on training time than the novices training to plateau. At every comparison point of scoring versus hands-on training time, novices in the mastery learning group on average consistently surpass those who train until plateau (Figure 4). The trainees in the plateau group improved their score but were not guided by any goal. Neither did they know what score could be obtainable for them or the gaps between their performance and expert performance. Within attribution theory of motivation, it is theorized that if an outcome is positive or expected, the learner is content while if the opposite is true, then the learner will search for an explanation for the result [18]. If the learners feel in control to alter the outcome they will be motivated to do so. In addition, learners may positively model their beliefs of self-efficacy based on the outcomes of others. Such a theory could explain our findings, as the novices training to plateau had no inkling that their performances were well below expert performance. They were simply not motivated to improve their performance, while the opposite occurred for the novices in the mastery learning group.

This study conveys supporting validity evidence for the simulation-based test and proposes a feasible and defensible pass/fail standard for novice trainees in orthopedic surgery. Nevertheless, caution should be taken not to interpret the study setup as optimal for learning in the simulated setting. The novices in the mastery learning group spent only 65 minutes on hands-on training. Such a brief period of training can hardly ensure optimal learning of a complex procedure. Indeed, it is known that performance during acquisition of skills can be an imperfect indicator of learning. For example, providing frequent information on performance errors during training can improve performance but be detrimental to the retention or transfer of skills [19]. Dyre et al. [20] examined the effect of error avoidance training (EAT) against error management training (EMT) on a vaginal ultrasound simulator. The EAT group was instructed to follow the simulator instructions exactly and aim for the highest possible simulator score while the EMT group was instructed to experiment and explore as much as they wanted and consider errors as positive and indicative of their learning. Both groups improved vastly between pre- and post-test on the simulator, but the EMT group outperformed the EAT group both statistically and clinically significant on a subsequent transfer test on patients in the clinical setting. It is reasonable to expect that these findings are transferable to SBT in osteosynthesis of PFF and exploration of optimal learning and transfer to the operating room in this setting is warranted. Moreover, residents should train when they have a demand to acquire the technical skill and have the opportunity to apply the skill in the clinical setting [21].

The metrics employed to calculate the test score (Table 1) was selected by 2 experts and deemed clinically relevant. Though this is a common practice during development of simulation-based tests, it incurs the risk of selecting metrics that fail to effectively distinguish between proficiency and its absence. If the total score encompasses many such non-discriminatory metrics the test will be less sensitive. In an exploration of the validity of a SBT bronchoscopy test the metric “segments visualized,” despite its high clinical relevance, did not effectively differentiate between novices and experts [22]. A closer examination of recorded performances unveiled that the novices indeed visualized all segments but did so akin to a rat in a maze in contrast to a structured performance by the experts. Consequently, while the metric is clinically significant, it proved to be an inadequate measure of competence on its own. Reduction in the sensitivity of test scores heightens the likelihood of encountering a “ceiling effect” in test scores, rendering it disproportionately easier for novices to attain high scores, as may be the case in this study. Another factor may be the inherent simplicity of the simulation-based testing environment. Unlike the complexities of real surgical settings, the simulator provides clear, uninterrupted fluoroscopic views without the common delays, such as those encountered while waiting for an assistant to switch between anteroposterior and lateral projections. Additionally, the simulator’s predefined steps mitigate the risk of some procedural errors, such as neglecting to rotate the set screw counterclockwise to allow for dynamic compression, further simplifying the test environment. The simulation also lacks the natural variation in bone anatomy and fracture types seen in actual cases and does not incorporate the often challenging process of fracture reduction, which can be vital for successful surgical intervention. Consequently, this uniformity in the simulation might limit its capacity to distinguish between more seasoned practitioners, such as 4th-year trainees, and experts [23]. This limitation could impact the test’s effectiveness in accurately assessing more advanced levels of surgical expertise.

A notable strength of the study lies in its focus on 1st-year trainees as novice participants, who represent the primary target group with minimal or no specific procedural practice under supervision.

However, the mere availability of SBT with pass/fail standards does not guarantee standardized training for all individuals in need. For example, SBT for osteosynthesis of PFF has been recommended in the Danish national curriculum for orthopedic surgery since 2022. Despite this, within the educational region served by the CAMES, there have been 69 1st-year positions available since the incorporation. Yet only 36 have participated in the introductory course, and 9 failed to train to the point of meeting the pass/fail criteria. Other specialties have reported comparable outcomes [24]. This phenomenon is likely attributable to multiple factors. Rölfing et al. [25] observed that for 1st-year orthopedic trainees who met the pass/fail criteria for an SHS procedure using the same simulator, the sensitivity for continuing in an orthopedic career 2 years post-assessment was 59%, with a specificity of 90%, indicating that intrinsic motivation can be a contributing factor. Other factors like protected training time, availability of training, implementation processes, and stakeholders’ attitudes are possibly just as important and further research into implementation of SBT is warranted [26]. Also, transfer of learning from the simulated setting to clinical practice is central to all SBT and future research into exploration of the consequences of test results on downstream performance of trainees is important to assess the impact of SBT on clinical practice, and to expand the sources of validity evidence, and the interpretation of test validity.

### Conclusion

Our study provides supporting validity evidence from all sources of Messick’s framework for a simulation-based test in short antegrade nail osteosynthesis of intertrochanteric hip fracture and establishes a defensible pass/fail standard for mastery learning SBT, relevant for novice orthopedic trainees prior to advancing to supervised training in the operating room. Novices who practiced using mastery learning were able to reach the pre-defined pass/fail standard and outperformed novices without a set goal for external motivation.

## Notes

AG: study design, data collection, statistical analysis, data analysis and interpretation, writing of the manuscript. JDR: study design, data collection, and revision of the manuscript. BV: study design, data collection, and revision of the manuscript. HP: study design and revision of the manuscript. SG: statistical analysis, data analysis, interpretation, and revision of the manuscript. LK: study design, statistical analysis, data analysis and interpretation, revision of the manuscript.

Handling co-editors: Cecilia Rogmark and Robin Christensen

*Acta* thanks Lars Enochsson and Chinmay Gupte for help with peer review of this manuscript.
